# An open, observational clinical study of neoadjuvant therapy in resectable stage III non-small cell lung cancer

**DOI:** 10.3389/fonc.2023.1194100

**Published:** 2023-08-16

**Authors:** Yuwen Qi, Linping Gu, Jie Shen, Yaxian Yao, Yi Zhao, Shun Lu, Zhiwei Chen

**Affiliations:** Department of Oncology, Shanghai Chest Hospital, Shanghai Jiao Tong University School of Medicine, Shanghai, China

**Keywords:** resectable stage III NSCLC, neoadjuvant therapy, disease-free survival, pathological complete response, clinical trial, adverse events, surgical feasibility

## Abstract

**Background:**

This open, observational clinical study aimed to investigate the efficacy, safety and survival outcomes of neoadjuvant chemotherapy, neoadjuvant immunotherapy with(out) chemotherapy and neoadjuvant targeted therapy among resectable stage III non-small cell lung cancer (NSCLC) patients (NCT04197076) in real world. 48 of the 57 evaluable patients were included in this interim analysis.

**Methods:**

This study was conducted at Shanghai Chest Hospital and included eligible NSCLC patients who were 18 years or older and had resectable clinical stage III disease. Surgical resection was conducted after neoadjuvant chemotherapy (13 patients), immunotherapy with(out) chemotherapy (26 patients), and targeted therapy (9 patients). Disease-free survival (DFS) was evaluated as the primary endpoint. The secondary endpoint was pathological complete response (pCR) rate. Clinical response rate (cRR), related adverse events (AEs), surgical feasibility and pathological features were also discussed in this study.

**Results:**

Significant differences in DFS were noted between chemotherapy and immunotherapy [7.7 months (range, 3.1 to 23.2 months) vs. 9.6 months (range, 4.0 to 47.9 months); P=0.032], and between chemotherapy and targeted therapy [7.7 months (range, 3.1 to 23.2 months) vs. 13.2 months (range, 7.5 to 32.2 months); P=0.015], but not between immunotherapy and targeted therapy (P=0.500). Subgroup analysis also favored neoadjuvant immunotherapy and targeted therapy. 5 patients achieved pathological complete response (pCR), all of whom were in the neoadjuvant immunotherapy arm, leading to a pCR rate of 19.2% in this arm. Treatment-emergent adverse events (TEAEs) of over grade 3 occurred in 11 patients (19.3%), with 5 (29.4%) in the chemotherapy arm, 5 (16.7%) in the immunotherapy arm and 1 (10.0%) in the targeted therapy arm. One grade 4 and one grade 2 surgery-related serious adverse event occurred in the neoadjuvant chemotherapy and immunotherapy arm, respectively.

**Conclusion:**

In patients diagnosed with resectable stage III NSCLC, neoadjuvant immunotherapy and neoadjuvant targeted therapy were associated with significantly longer disease-free survival compared with neoadjuvant chemotherapy. Clinical and pathological response rates were also higher in the immunotherapy and targeted therapy arm. Adverse events were found to be manageable and similar across all three groups, and surgical feasibility favored immunotherapy or targeted therapy rather than chemotherapy.

**Clinical trial registration:**

https://clinicaltrials.gov/, identifier NCT04197076.

## 1 Introduction

Lung cancer, with non-small cell lung cancer (NSCLC) accounting for approximately 85% of cases, remains the leading cause of cancer death (1, 2). At diagnosis, about 1/3 of NSCLC cases are presented at stage III, which typically requires multimodality treatment incorporating systemic neoadjuvant therapy, surgery and adjuvant therapy (3, 4).

Neoadjuvant therapy aims to reduce tumor size and burden, while acting on occult micro-metastatic disease, thereby increasing the likelihood of downstaging and complete surgical resection, as well as reducing the risk of recurrence (5). Previous studies have demonstrated that neoadjuvant therapy could be a cornerstone towards improved efficacy and survival for patients (6–8).

The most widely applied neoadjuvant therapies include neoadjuvant chemotherapy, neoadjuvant immunotherapy and neoadjuvant targeted therapy. Apart from efficacy, the phase II LCMC3 trial also showed the safety and promising survival of neoadjuvant immunotherapy in patients with resectable stage IB-IIIB NSCLC (9), while another phase II study demonstrated that neoadjuvant therapy with gefitinib appeared to be safe and effective in patients with stage II-IIIA NSCLC (7).

Moreover, compared to neoadjuvant chemotherapy, neoadjuvant immunotherapy and targeted therapy have better overall results in patients with resectable NSCLC as suggested by the Checkmate 816 trial and the EMERGING-CTONG 1103 trial, respectively (10, 11).

Prompted by these encouraging outcomes, we carried out this interim analysis of study to investigate the efficacy, safety and survival of different neoadjuvant therapies, including neoadjuvant chemotherapy, neoadjuvant immunotherapy with(out) chemotherapy, and neoadjuvant targeted therapy in patients with resectable stage III NSCLC in real world.

## 2 Methods

### 2.1 Study design and participants

This open, observational clinical study of neoadjuvant chemotherapy, immunotherapy with(out) chemotherapy and targeted therapy for resectable stage III NSCLC was conducted at Shanghai Chest Hospital. Eligible patients had Eastern Cooperative Oncology Group (ECOG) performance status 0 or 1, were 18 years or older, had not received antitumor treatment for stage III NSCLC confirmed by a Positron Emission Tomography-computed tomography (PET-CT)/a whole body bone scan along with a contrast-enhanced CT scan and cranial magnetic resonance imaging (MRI) according to the 8th International Association for the Study of Lung Cancer (IASLC) that was considered surgically operable by certified thoracic surgeons, had at least one measurable lesion according to Response Evaluation Criteria In Solid Tumors Version 1.1 (RECIST 1.1), and had tumor samples that could be used for gene detection and Programmed Cell Death-Ligand 1 (PD-L1) immunohistochemistry (IHC) examination.

This study was registered at ClinicalTrials.gov [NCT04197076]. Full inclusion and exclusion criteria are included in the trial protocol approved by the institutional review board. Patients had the right to withdraw from the trial for any reason at any time, and researchers had the right to withdraw patients from the study due to intolerant toxicity, protocol violation, or other reasons. The trial was performed according to the International Conference on Harmonization Good Clinical Practice guidelines. According to the Declaration of Helsinki, all patients signed informed consent before participating.

### 2.2 Treatment procedures

Patients with epidermal growth factor receptor (EGFR) mutation, anaplastic lymphoma kinase (ALK) translocation, and ROS proto-oncogene 1 receptor tyrosine kinase (ROS-1) rearrangement were offered an appropriate tyrosine kinase inhibitor including afatinib, gefitinib, or crizotinib. Afatinib and gefitinib were used for EGFR mutation and patients with ALK translocation or ROS-1 rearrangement were given crizotinib. Patients detected with PD-L1 were enrolled from the Checkmate 816 trial (immunotherapy ± chemotherapy) or immunotherapy (checkpoint inhibitors). Immunotherapeutic agents were selected among nivolumab, pembrolizumab, sintilimab, tislelizumab (PD-1 inhibitors), and ipilimumab (CTLA-4 inhibitor). Other patients were assigned chemotherapy alone. Different chemotherapy regimens were adopted according to the clinical characteristics following National Comprehensive Cancer Network (NCCN) guidelines.

All patients were reviewed for response to therapy at the end of treatment according to RECIST 1.1, with surgical intervention scheduled within 6 weeks. Clinicopathological data were retrieved from electronic medical records and pathologic reports. The level of TILs was evaluated as a percentage in 10% increments, with a 1% or 5% criteria utilized when TILs level was less than 10%. As no standardized cutoff for TILs level was provided (12), patients were divided into 2 categories base on TILs level for statistical analysis purposes: ≥30% and <30%. Following surgical intervention, necessary radiotherapy, chemotherapy, immunotherapy, or targeted therapy were administered according to NCCN guidelines, and adverse events were evaluated according to common terminology criteria for adverse events (CTCAE) v5.0.

Patients are being followed up at 3 months post-treatment, and every 3 months afterwards for the first 2–3 years, every 4–6 months for an additional 2 years, and annually thereafter. Follow-up assessments included a physical examination, complete blood count, blood biochemistry, tumor marker, thoracic CT scan, abdomen B-ultrasound examination, and enhanced CT or magnetic resonance imaging (MRI) examination of suspected lesions.

### 2.3 Endpoints

The primary endpoint of this study was disease-free survival (DFS), defined as the time from the receipt of pathological and genetic diagnosis reports to the occurrence of any of the following disease progression, disease recurrence (according to RECIST 1.1), or death from any cause.

The secondary endpoint was pathological complete response (pCR) rate, which was defined as the proportion of patients who achieved pathologic complete response (lack of all signs of tumor in tissue samples removed during surgery after treatment).

Exploratory objectives and endpoints included clinical response rate (cRR), related adverse events (AEs), surgical feasibility and pathological features.

### 2.4 Statistical analysis

Statistical analysis was conducted using the full analysis set, which included all evaluable patients. Two-tailed test, two-way ANOVA, chi-squared test, Kaplan-Meier survival analyses, log-rank test and Mann-Whitney U test were used as appropriate. Continuous variables were described as mean with standard deviation (SD) or median with range. All statistical analyses were performed using SPSS (IBM SPSS Statistics 25) and R V.4.2.2, with a two-sided P value of less than 0.05 indicating a statistically significant difference.

## 3 Results

### 3.1 Baseline characteristics

**Figure 1:**
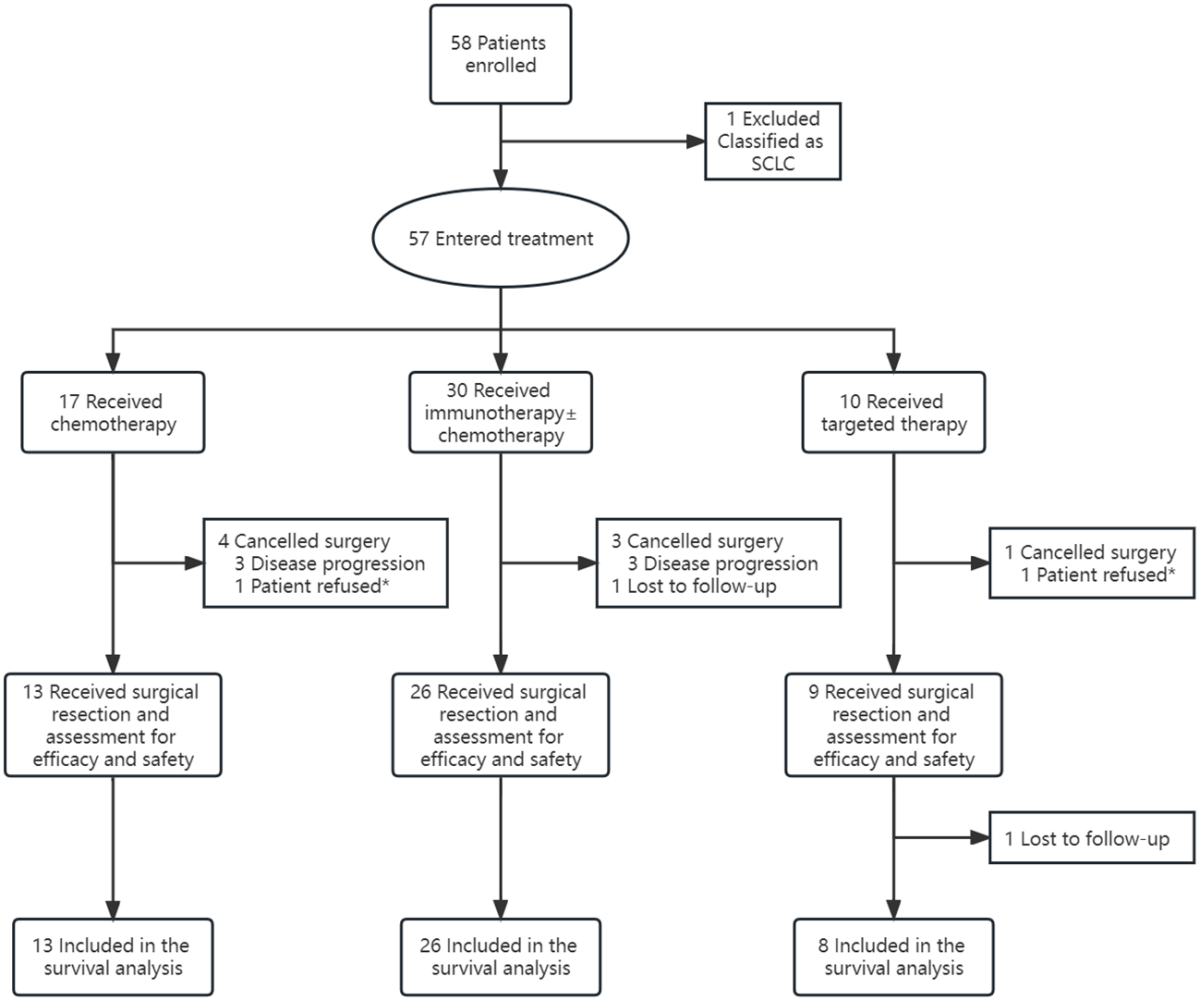
Study Profile. * The patients achieved tumor regression according to computed tomographic (CT) scans and preferred a conservative therapeutic approach.

From September 2018 to July 2022, a total of 58 patients presenting with resectable stage III NSCLC were enrolled for neoadjuvant therapy, with the exception of one patient who was later diagnosed with small cell lung cancer (SCLC) through pathological confirmation and thus excluded from the study. Amongst the remaining 57 patients, 17 (29.8%) received chemotherapy (including three patients from Checkmate 816), 30 (52.6%) received immunotherapy [26 patients received immunotherapy + chemotherapy (2 from Checkmate 816), another 2 patients from Checkmate 816 received nivolumab + ipilimumab, one patient received pembrolizumab alone, and one patient received sintilimab alone], and 10 (17.5%) received targeted therapy (7 patients with EGFR 19del/21L858R mutations, 2 patients with EML4-ALK translocations, and one patient with other genetic mutations). 48 of all enrolled patients underwent surgical resections [disease progression (n=6); lost to follow-up (n=1); patient refusal (n=2)], with 13 (27.1%) in the chemotherapy arm, 26 (54.2%) in the immunotherapy arm, and 9 (18.8%) in the targeted therapy arm (**Figure 1**).

Before the data cutoff date (March 4, 2023), another patient discontinued the study (lost to follow-up), leaving 47 patients evaluable for survival analysis. **Table 1** provides an overview of the baseline characteristics of these patients.

**Table 1:** Baseline characteristics of all patients (n=57) and patients in different neoadjuvant therapy groups.

Characteristics	All n=57 (%)	Chemotherapy n=17 (%)	Immunotherapy n=30 (%)	Targeted therapy n=10 (%)
Age (years), mean ± SD	62.5 ± 8.9	67.4 ± 6.9	62.3 ± 8.5	54.7 ± 7.9
BMI (kg/m2), mean ± SD	24.3 ± 2.5	24.5 ± 2.6	24.2 ± 2.7	24.3 ± 2.0
Gender				
Male	43 (75.4)	13 (76.5)	27 (90.0)	3 (30.0)
Female	14 (24.6)	4 (23.5)	3 (10.0)	7 (70.0)
ECOG performance status				
0	5 (8.8)	0 (0)	3 (10.0)	2 (20.0)
1	52 (91.2)	17 (100.0)	27 (90.0)	8 (80.0)
Smoking status				
Yes/Ever	30 (52.6)	11 (64.7)	18 (60.0)	1 (10.0)
Never	27 (47.4)	6 (35.3)	12 (40.0)	9 (90.0)
Histology				
SCC	32 (56.1)	12 (70.6)	19 (63.3)	1 (10.0)
ADC	21 (36.8)	5 (29.4)	8 (26.7)	8 (80.0)
Not specified	4 (7)	0 (0)	3 (10)	1 (10.0)
cT-TNM8				
T1b	1 (1.8)	1 (5.9)	0 (0)	0 (0)
T2a	18 (31.6)	3 (17.6)	8 (26.7)	7 (70.0)
T2b	9 (15.8)	3 (17.6)	5 (16.7)	1 (10.0)
T3	15 (26.3)	5 (29.4)	8 (26.7)	2 (20.0)
T4	14 (24.6)	5 (29.4)	9 (30.0)	0 (0)
cN-TNM8				
N0	2 (3.5)	1 (5.9)	1 (3.3)	0 (0)
N1	6 (10.5)	2 (11.8)	4 (13.3)	0 (0)
N2	46 (80.7)	13 (76.5)	23 (76.7)	10 (100.0)
N3	3 (5.3)	1 (5.9)	2 (6.7)	0 (0)
TNM8				
III A	34 (59.6)	10 (58.8)	16 (53.3)	8 (80.0)
III B	22 (38.6)	6 (35.3)	14 (46.7)	2 (20.0)
III C	1 (1.8)	1 (5.9)	0 (0)	0 (0)
Gene status				
Wild-type	22 (38.6)	9 (52.9)	13 (43.3)	0 (0)
EGFR mutation	8 (14)	1 (5.9)	0 (0)	7 (70.0)
ALK translocation	3 (5.3)	1 (5.9)	0 (0)	2 (20.0)
ROS-1 rearrangement	0 (0)	0 (0)	0 (0)	0 (0)
Other	24 (42.1)	6 (35.3)	17 (56.7)	1 (10.0)
PD-L1 expression				
<1%	20 (35.1)	10 (58.8)	9 (30.0)	1 (10.0)
1%-50%	10 (17.5)	2 (11.8)	7 (23.3)	1 (10.0)
>50%	10 (17.5)	2 (11.8)	6 (20.0)	2 (20.0)
Unknown	17 (29.8)	3 (17.6)	8 (26.7)	6 (60)

SD, standard deviation; BMI, body mass index; ECOG, Eastern Cooperative Oncology Group; ADC, adenocarcinoma; SCC, squamous cell carcinoma; cT-TNM8, clinical T stage according to TNM eighth edition; cN-TNM8, clinical N stage according to TNM eighth edition; TNM8, stage according to TNM eighth edition.

### 3.2 Clinical response

**Figure 2:**
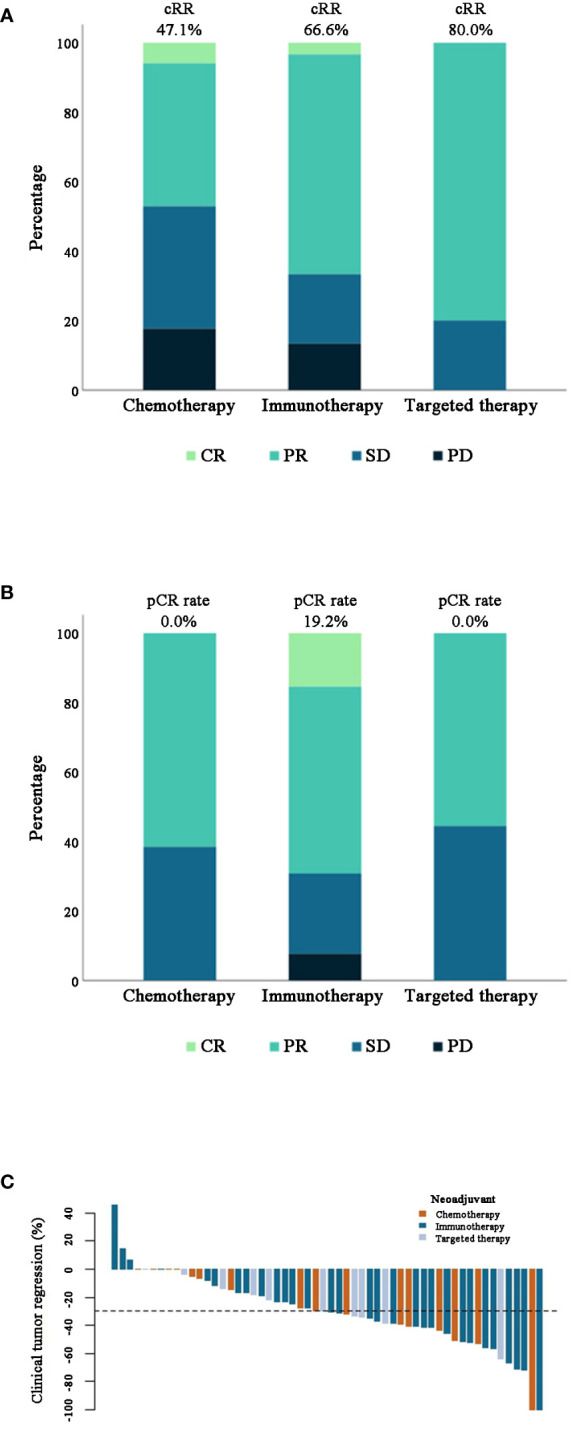
**(A, B)** Stacked bar plots of clinical (n=57) and pathological (n=48) response rate of patients in different treatment groups. **(C)** Waterfall plot of clinical tumor regression percentage after different neoadjuvant therapies in 56 patients. Note: One patient in the chemotherapy group was excluded as mentioned before.

Upon completion of the designated neoadjuvant therapy, one patient (5.9%) in the chemotherapy arm and one patient (3.3%) in the immunotherapy arm attained clinical complete response (CR). Meanwhile, partial response (PR) was achieved by 7 patients (41.2%) in the chemotherapy arm, 19 patients (63.3%) in the immunotherapy arm and 8 patients (80.0%) in the targeted therapy arm, resulting in a cRR of 47.1%, 66.6%, and 80.0% in each treatment arm (**Table 2**, **Figure 2A**). No significant differences were detected among different therapy arms (P=0.358). The clinical tumor regression of all patients (n=57) was depicted in **Figure 2C**.

**Table 2:** Clinical (n=57) and pathological (n=48) response of all patients and patients in different neoadjuvant therapy group.

	All n=57 (%)	Chemotherapy n=17 (%)	Immunotherapy n=30 (%)	Targeted therapy n=10 (%)	*P*-value
Clinical response					0.358^*^
CR	2 (3.5)	1 (5.9)	1 (3.3)	0 (0)	
PR	34 (59.6)	7 (41.2)	19 (63.3)	8 (80.0)	
SD	14 (24.6)	6 (35.3)	6 (20)	2 (20.0)	
PD	7 (12.3)	3 (17.6)	4 (13.3)	0 (0)	
Clinical tumor regression (%)					0.734
Mean ± SD	29.9 ± 26.6^a^	27.8 ± 27.3^a^	32.5 ± 28.8	25.7 ± 18.7	
(range)	(-45.7-100.0)	(0.0-100.0)	(-45.7-100.0)	(0.0-64.0)	
	n=48 (%)	n=13 (%)	n=26 (%)	n=9 (%)	
Pathological response					0.122^*^
CR	5 (10.4)	0 (0)	5 (19.2)	0 (0)	
PR	26 (54.2)	8 (61.5)	13 (50.0)	5 (55.6)	
SD	15 (31.3)	5 (38.5)	6 (23.1)	4 (44.4)	
PD	2 (4.2)	0 (0)	2 (7.7)	0 (0)	

CR, complete response; PR, partial response; SD, stable disease; PD, progressive disease.

^*^likelihood ratio.

a One patient in the chemotherapy arm was reported to have disease progression by the investigator, but precise measurement of the lesions was impeded by the infiltrative tumor border configuration. The patient was therefore excluded from the analysis of tumor regression.

### 3.3 Surgery

As illustrated in **Figure 1**, among the patients who entered treatment, 4 patients (23.5%) in the chemotherapy arm (3 due to disease progression and 1 due to patient refusal), 3 patients (10.0%) in the immunotherapy arm (all due to disease progression), and one patient (10.0%) in the targeted therapy arm (due to patient refusal) cancelled surgery. Additionally, one patient in the immunotherapy group was lost to follow-up. The remaining 48 eligible patients all underwent surgery. One patient (7.7%) in the chemotherapy arm delayed surgery because of an adverse event (hemoptysis), 3 patients (11.5%) in the immunotherapy arm delayed surgery owing to the pandemic, smoking cessation of less than two weeks and the involvement in a car accident, and one patient (11.1%) in the targeted therapy arm delayed surgery due to drug withdrawal of less than two weeks. The median time of delay was 2.0 weeks overall (**Table 3**).

**Table 3:** Surgical outcomes of patients in different treatment arms.

Surgical outcomes	All n=48 (%)	Chemotherapy n=13 (%)	Immunotherapy n=26 (%)	Targeted therapy n=9 (%)	*P*-value
Surgical cancellation	8 (14.0)	4 (23.5)	3 (10.0)	1 (10.0)	
Surgical delay^a^	5 (10.4)	1 (7.7)	3 (11.5)	1 (11.1)	
Median time (w)(range)	2.0	4.0	2.0 (2.0-8.0)	2.0	
Adverse event(s)	1 (2.1)	1 (7.7)	0 (0)	0 (0)	
Tumor location					0.696^*^
LUL	12 (25)	5 (38.5)	5 (19.2)	2 (22.2)	
LLL	6 (12.5)	1 (7.7)	4 (15.4)	1 (11.1)	
RUL	21 (43.8)	5 (38.5)	13 (50)	3 (33.3)	
RML	2 (4.2)	1 (7.7)	1 (3.8)	0 (0)	
RLL	7 (14.6)	1 (7.7)	3 (11.5)	3 (33.3)	
Approach					0.008^*^
VATS	30 (62.5)	7 (53.8)	14 (53.8)	9 (100)	
Thoracotomy	18 (37.5)	6 (46.2)	12 (46.2)	0 (0)	
Type					0.348^*^
Segmentectomy	2 (4.2)	0 (0)	1 (3.8)	1 (11.1)	
Lobectomy	19 (39.6)	12 (92.3)	25 (96.2)	8 (88.9)	
Pneumonectomy	1 (2.1)	1 (7.7)	0 (0)	0 (0)	
Duration of surgery (min), median (range)	90.0 (60.0-270.0)	90.0 (60.0-210.0)	90.0 (60.0-210.0)	60.0(60.0-270.0)	0.134^*^
≤120min	30 (62.5)	8 (61.5)	14 (53.8)	8 (88.9)	
>120min	18 (37.5)	5 (38.5)	12 (46.2)	1 (11.1)	
Amount of bleeding (ml), median (range)	100.0 (20.0-300.0)	100.0 (50.0-300.0)	100.0 (20.0-200.0)	100.0 (100.0-300.0)	0.240^*^
≤100 ml	32 (66.7)	8 (61.5)	16 (61.5)	8 (88.9)	
>100 ml	16 (33.3)	5 (38.5)	10 (38.5)	1 (11.1)	
Surgical margin					0.356^*^
R0	45 (93.8)	13 (100)	24 (92.3)	8 (88.9)	
R+	3 (6.3)	0 (0)	2 (7.7)	1 (11.1)	
No. of drainage tubes					0.843^*^
1	15 (31.3)	4 (30.8)	8 (30.8)	3 (33.3)	
2	33 (68.8)	9 (69.2)	18 (69.2)	6 (66.7)	
No. of ICU transfer,	20 (41.7)	5 (38.5)	13 (50.0)	2 (22.2)	
median time (d) (range)	1 (1-10)	3 (1-10)	1 (1-3)	1 (1-1)	
Length of stay (d), median (range)	6 (3-31)	6 (3-15)	6.5 (3-31)	4 (3-7)	0.120
≤6d	29 (60.4)	8 (61.5)	13 (50.0)	8 (88.9)	
>6d	19 (39.6)	5 (38.5)	13 (50.0)	1 (11.1)	
pT-TNM8					0.003^*^
T0	5 (10.4)	0 (0)	5 (19.2)	0 (0)	
T1b	4 (8.3)	0 (0)	2 (7.7)	2 (22.2)	
T1c	9 (18.8)	1 (7.7)	7 (26.9)	1 (11.1)	
T2a	12 (25)	8 (61.5)	4 (15.4)	0 (0)	
T2b	10 (20.8)	1 (7.7)	4 (15.4)	5 (55.6)	
T3	5 (10.4)	2 (15.4)	2 (7.7)	1 (11.1)	
T4	3 (6.3)	1 (7.7)	2 (7.7)	0 (0)	
pN-TNM8					0.314^*^
N0	19 (39.6)	3 (23.1)	14 (53.8)	2 (22.2)	
N1	6 (12.5)	2 (15.4)	2 (7.7)	2 (22.2)	
N2	22 (45.8)	8 (61.5)	9 (34.6)	5 (55.6)	
N3	1 (2.1)	0 (0)	1 (3.8)	0 (0)	
p-TNM8					0.014^*^
Stage 0	5 (10.4)	0 (0)	5 (19.2)	0 (0)	
IA	5 (10.4)	0 (0)	5 (19.2)	0 (0)	
IB	6 (12.5)	2 (15.4)	2 (7.7)	2 (22.2)	
IIA	3 (6.3)	0 (0)	1 (3.8)	2 (22.2)	
IIB	6 (12.5)	3 (23.1)	3 (11.5)	0 (0)	
IIIA	16 (33.3)	7 (53.8)	5 (19.2)	4 (44.4)	
IIIB	7 (14.6)	1 (7.7)	5 (19.2)	1 (11.1)	
Pathological downstaging					
T stage					0.069^*^
Yes	35 (72.9)	9 (69.2)	22 (84.6)	4 (44.4)	
No	13 (27.1)	4 (30.8)	4 (15.4)	5 (55.6)	
N stage					0.843^*^
Yes	24 (50)	6 (46.2)	14 (53.8)	4 (44.4)	
No	24 (50)	7 (53.8)	12 (46.2)	5 (55.6)	
TNM stage					0.736^*^
Yes	31 (64.6)	8 (61.5)	18 (69.2)	5 (55.6)	
No	17 (35.4)	5 (38.5)	8 (30.8)	4 (44.4)	

LUL, left upper lobe; LLL, left lower lobe; RUL, right upper lobe; RML, right middle lobe; RLL, right lower lobe; VATS, video-assisted thoracic surgery; No., number; pT-TNM8, pT stage according to TNM eighth edition; pN-TNM8, pN stage according to TNM eighth edition;

^*^likelihood ratio.

a The patient in the chemotherapy arm experienced hemoptysis shortly after treatment, and subsequently received embolization at a local hospital.

The bold values denote statistical significance at P<0.05 level.

The rate of patients who underwent video-assisted thoracic surgery (VATS) was roughly the same between the chemotherapy arm and the immunotherapy arm, but significantly higher in the targeted therapy arm (P=0.008). Most patients underwent lobectomy, while 2 patients (one each in the immunotherapy and targeted therapy arms) underwent segmentectomy and one patient in the chemotherapy arm received pneumonectomy. The median duration of surgery and the amount of bleeding were 90 minutes and 100ml, respectively. Only 3 (6.3%) did not achieve complete tumor resection (R0) [2 (7.7%) in the immunotherapy arm and 1 (10.0%) in the targeted therapy arm] among the 48 patients who underwent surgery. No significant differences were observed among the different treatment arms.

A total of 31 patients experienced clinical to pathological downstaging, including 8 (61.5%) in the chemotherapy arm, 18 (69.2%) in the immunotherapy arm and 5 (55.6%) in the targeted therapy arm. Additional surgical outcomes can be found in **Table 3**.

### 3.4 Pathological response and features

**Figure 3:**
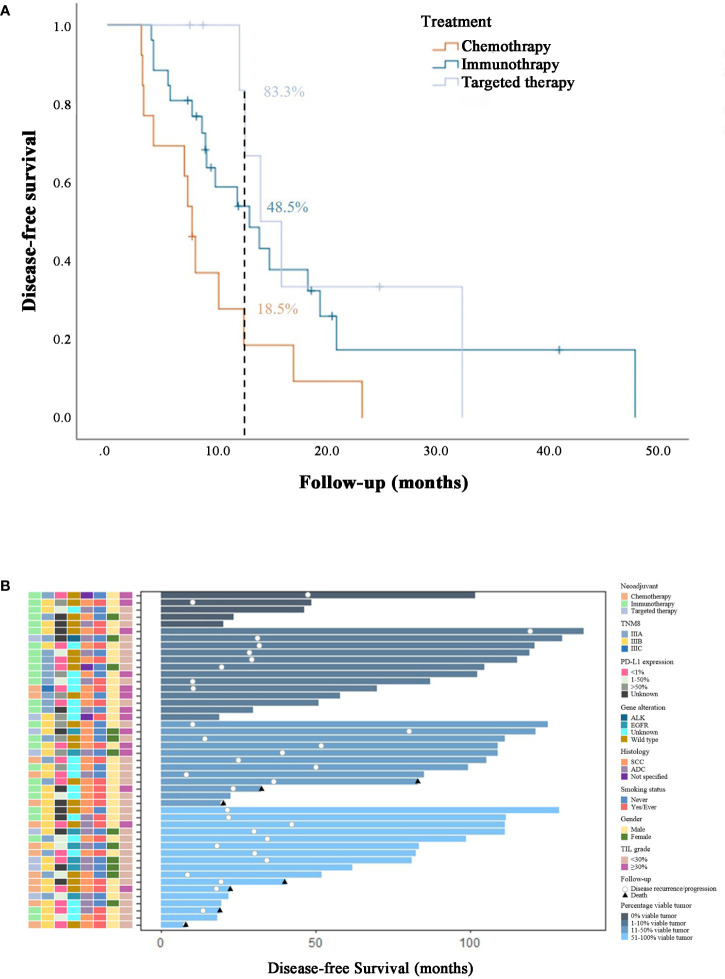
**(A)** Survival analysis in stage III NSCLC patients from different neoadjuvant therapy groups. **(B)** Swimmer plot of disease-free survival in patients who underwent surgical resection and were evaluable (n=47), with each bar representing an individual patient. Clinical features were displayed on the left. As of the data cutoff date on Mar.4, 2023, 12 (25.5%) patients had not yet met the primary endpoint. Among the remaining patients who did reach disease-free survival, 7 patients had died, 4 of whom were from the neoadjuvant chemotherapy arm, and the other 3 were from the immunotherapy arm.

5 people achieved pCR and all of them were in the immunotherapy arm, bringing the pCR rate of this arm to 19.2% (**Table 2**, **Figure 2B**). Based on the analysis of TILs from surgically resected specimens, 33 patients (68.8%) were categorized as having a low TILs level (<30%), whereas the other 15 patients (31.3%) had a high TILs level (≥30%). A significantly higher rate of patients in the immunotherapy group had foamy macrophages in their specimens (P=0.018). Other pathological features of these patients were shown in **Table 4**. The characteristics of 47 patients in different percentage viable tumor groups are shown in **Figure 3B** (one patient was lost to follow-up later).

**Table 4:** Pathological features of resected specimens from patients who underwent surgery.

Pathological features	All n=48 (%)	Chemotherapy n=13 (%)	Immunotherapy n=26 (%)	Targeted therapy n=9 (%)	*P*-value
Tumor infiltrating lyphocytes^a^					0.631^*^
<30%	33 (68.8)	9 (69.2)	19 (73.1)	5 (55.6)	
≥30%	15 (31.3)	4 (30.8)	7 (26.9)	4 (44.4)	
Dense plasma cells					0.661^*^
Absent	34 (70.8)	10 (76.9)	17 (65.4)	7 (77.8)	
Present	14 (29.2)	3 (23.1)	9 (34.6)	2 (22.2)	
Cholesterol clefts					0.616^*^
Absent	41 (85.4)	12 (92.3)	22 (84.6)	7 (77.8)	
Present	7 (14.6)	1 (7.7)	4 (15.4)	2 (22.2)	
Foamy macrophages					0.018^*^
Absent	33 (68.8)	13 (100)	20 (76.9)	9 (100)	
Present	6 (12.5)	0 (0)	6 (23.1)	0 (0)	
Proliferative fibrosis					0.498^*^
Absent	10 (20.8)	2 (15.4)	7 (26.9)	1 (11.1)	
Present	38 (79.2)	11 (84.6)	19 (73.1)	8 (88.9)	
Hyalinization					0.739^*^
Absent	40 (83.3)	10 (76.9)	22 (84.6)	8 (88.9)	
Present	8 (16.7)	3 (23.1)	4 (15.4)	1 (11.1)	
Giant cells					0.589^*^
Absent	37 (77.1)	10 (76.9)	19 (73.1)	8 (88.9)	
Present	11 (22.9)	3 (23.1)	7 (26.9)	1 (11.1)	
Necrosis					0.324
Absent	29 (60.4)	6 (46.2)	16 (61.5)	7 (77.8)	
Present	19 (39.6)	7 (53.8)	10 (38.5)	2 (22.2)	

^*^likelihood ratio.

The bold values denote statistical significance at P<0.05 level.

### 3.5 Adverse events

Of all 57 patients, 37 (64.9%) experienced at least one treatment-emergent adverse event (TEAE) of any grade, with myelosuppression being the most common (23/57, 40.4%). TEAEs of grade 3 or higher occurred in 11 patients (19.3%): 5 (29.4%) in the chemotherapy group, 5 (16.7%) in the immunotherapy group and 1 (10.0%) in the targeted therapy group. 30 patients experienced adverse events related to treatment, with 10 (58.8%) in the chemotherapy group, 16 (53.3%) in the immunotherapy group, and 4 (40.0%) in the targeted therapy group. The rates of treatment-related adverse events (TRAEs) of grade 3 or higher were 29.4%, 16.7%, and 10.0%, respectively. The rates of serious adverse events were similar in patients who received chemotherapy, immunotherapy and targeted therapy (**Table 5**). No adverse event led to discontinuation of treatment, and the toxicity was overall manageable.

**Table 5:** Treatment-emergent adverse events occurred during the neoadjuvant treatment process in all patients (n=57) and surgery-related adverse events occurred within 90 days after surgery in patients who underwent surgical resection (n=48).

Treatment-emergent adverse events	All n=57 (%)		Chemotherapy n=17 (%)		Immunotherapy n=30 (%)		Targeted therapy n=10 (%)	
	Any grade	Grade ≥3	Any grade	Grade ≥3	Any grade	Grade ≥3	Any grade	Grade ≥3
	Number of patients (%)							
Any event	37 (64.9)	11 (19.3)	10 (58.8)	5 (29.4)	21 (70.0)	5 (16.7)	6 (60.0)	1 (10.0)
Event leading to discontinuation of treatment	0 (0)	0 (0)	0 (0)	0 (0)	0 (0)	0 (0)	0 (0)	0 (0)
SAE	6 (10.5)	4 (7.0)	2 (11.8)	1 (5.9)	3 (10.0)	2 (6.7)	1 (10.0)	1 (10.0)
Any TRAE	30 (52.6)	11 (19.3)	10 (58.8)	5 (29.4)	16 (53.3)	5 (16.7)	4 (40.0)	1 (10.0)
Treatment-related SAE	6 (10.5)	4 (7.0)	2 (11.8)	1 (5.9)	3 (10.0)	2 (6.7)	1 (10.0)	1 (10.0)
Event occurring in more than 1 patient								
Myelosuppression	23 (40.4)	9 (15.8)	7 (41.2)	5 (29.4)	13 (43.3)	3 (10)	3 (30.0)	1 (10.0)
Liver dysfunction	8 (14)	0 (0)	3 (17.6)	0 (0)	3 (10)	0 (0)	2 (20.0)	0 (0)
Rash	5 (8.8)	1 (1.8)	1 (5.9)	1 (5.9)	2 (6.7)	0 (0)	2 (20.0)	0 (0)
Vomiting	4 (7)	2 (3.5)	1 (5.9)	0 (0)	2 (6.7)	1 (3.3)	1 (10.0)	1 (10.0)
Anemia	4 (7)	0 (0)	3 (17.6)	0 (0)	1 (3.3)	0 (0)	0 (0)	0 (0)
Diarrhea	3 (5.3)	1 (1.8)	1 (5.9)	1 (5.9)	2 (6.7)	0 (0)	(0)	(0)
leucopenia	2 (3.5)	1 (1.8)	1 (5.9)	0 (0)	1 (3.3)	1 (3.3)^a^	0 (0)	0 (0)
Pain	2 (3.5)	0 (0)	0 (0)	0 (0)	2 (6.7)	0 (0)	0 (0)	0 (0)
Nausea	2 (3.5)	0 (0)	0 (0)	0 (0)	1 (3.3)	0 (0)	1 (10.0)	0 (0)
Decreased appetite	2 (3.5)	0 (0)	0 (0)	0 (0)	2 (6.7)	0 (0)	0 (0)	0 (0)
Fatigue	2 (3.5)	0 (0)	0 (0)	0 (0)	2 (6.7)	0 (0)	0 (0)	0 (0)
Radiation Pneumonitis	2 (3.5)	0 (0)	0 (0)	0 (0)	1 (3.3)	0 (0)	1 (10)	0 (0)
Peripheral neuropathy	2 (3.5)	0 (0)	1 (5.9)	0 (0)	1 (3.3)	0 (0)	0 (0)	0 (0)
Oral ulceration	2 (3.5)	0 (0)	0 (0)	0 (0)	1 (3.3)	0 (0)	1 (10)	0 (0)
Paronychia	2 (3.5)	0 (0)	0 (0)	0 (0)	1 (3.3)	0 (0)	1 (10)	0 (0)
Immune-related pneumonitis	1 (1.8)	0 (0)	0 (0)	0 (0)	1 (3.3)	0 (0)	0 (0)	0 (0)
Surgery-related adverse events	n=48 (%)		n=13 (%)		n=26 (%)		n=9 (%)	
Surgery-related SAE	2 (4.2)	1 (2.1)	1 (7.7)	1 (7.7)	1 (3.8)	0 (0)	0 (0)	0 (0)
Any surgery-related adverse events	25 (52.1)	1 (2.1)	8 (61.5)	1 (7.7)	14 (53.8)	0 (0)	3 (33.3)	0 (0)
Hydropneumothorax	15 (31.3)	0 (0)	4 (30.8)	0 (0)	10 (38.5)	0 (0)	1 (11.1)	0 (0)
Subcutaneous emphysema	14 (29.2)	0 (0)	4 (30.8)	0 (0)	8 (30.8)	0 (0)	2 (22.2)	0 (0)
Pleural effusion	5 (10.4)	0 (0)	2 (15.4)	0 (0)	3 (11.5)	0 (0)	0 (0)	0 (0)
Compressive atelectasis	2 (4.2)	0 (0)	1 (7.7)	0 (0)	1 (3.8)	0 (0)	0 (0)	0 (0)
Pneumothorax	1 (2.1)	0 (0)	0 (0)	0 (0)	1 (3.8)	0 (0)	0 (0)	0 (0)
Anemia	1 (2.1)	0 (0)	1 (7.7)	0 (0)	0 (0)	0 (0)	0 (0)	0 (0)
Pericardial effusion	1 (2.1)	0 (0)	0 (0)	0 (0)	1 (3.8)	0 (0)	0 (0)	0 (0)
Sinus tachycardia	1 (2.1)	0 (0)	0 (0)	0 (0)	1 (3.8)	0 (0)	0 (0)	0 (0)
Pain	1 (2.1)	0 (0)	0 (0)	0 (0)	1 (3.8)	0 (0)	0 (0)	0 (0)
Infection	1 (2.1)	0 (0)	0 (0)	0 (0)	0 (0)	0 (0)	1 (11.1)	0 (0)
Dyspnea	0 (0)	1 (2.1)	0 (0)	1 (7.7)^a^	0 (0)	0 (0)	0 (0)	0 (0)
Chyle leak	1 (2.1)	0 (0)	0 (0)	0 (0)	1 (3.8)^b^	0 (0)	0 (0)	0 (0)

TRAE, treatment-related adverse events; SAE, serious adverse event.

a This patient experienced sudden chest tightness and dyspnea on the second day after he was out of the ICU post-surgery, prompting immediate transfer back to the ICU with suspected pulmonary embolism. After spending another three days in the ICU with intermittent non-invasive ventilation, the patient’s general condition gradually improved and he was scheduled to be discharged the following week. However, he eventually died within two weeks after surgery at home and no further information could be obtained.

b This patient was diagnosed with chyle leak two days after surgery, and received appropriate treatment. As a result, his length of stay was prolonged.

Regarding patients who underwent surgical resection, 25/48 (52.1%) experienced surgery-related adverse events, mostly hydropneumothorax and/or subcutaneous emphysema. 8 (61.5%) of those patients were in the chemotherapy group, while 14 (53.8%) were in the immunotherapy group, and 3 (33.3%) were in the targeted therapy group. There were 2 cases of serious adverse events related to surgery. One was a grade 2 chyle leak in the immunotherapy group, and the other was a grade 4 dyspnea in the chemotherapy group. The patient in the chemotherapy group eventually died two weeks after surgery at home. Other surgical outcomes can be found in **Table 5**.

### 3.6 Survival analysis and follow-up

With 35 events of disease progression, recurrence or death, the median disease-free survival (DFS) was 9.4 months (range, 3.1 to 47.9 months) in the entire cohort of 47 evaluable patients. In the chemotherapy, immunotherapy and targeted therapy arms, the median DFS was 7.7 months (range, 3.1 to 23.2 months), 9.6 months (range, 4.0 to 47.9 months), and 13.2 months (range, 7.5 to 32.2 months), respectively. The estimated percentage of patients surviving without disease progression, recurrence, or death at 1 year was 18.5% in the chemotherapy arm, 48.5% in the immunotherapy arm and 83.3% in the targeted therapy arm. The Kaplan-Meier survival analysis (**Figure 3A**) revealed significant differences in DFS between the chemotherapy arm and the immunotherapy arm (P=0.032) and between the chemotherapy arm and the targeted therapy arm (P=0.015). There was no significant difference between the immunotherapy and targeted therapy arm (P=0.500).

As of the data cutoff date (March 4, 2023), 7 patients had died, with 4 of them (30.8%) in the chemotherapy arm and the other 3 (15.4%) in the immunotherapy arm (**Figure 3B**).

### 3.7 Subgroup analysis

**Figure 4:**
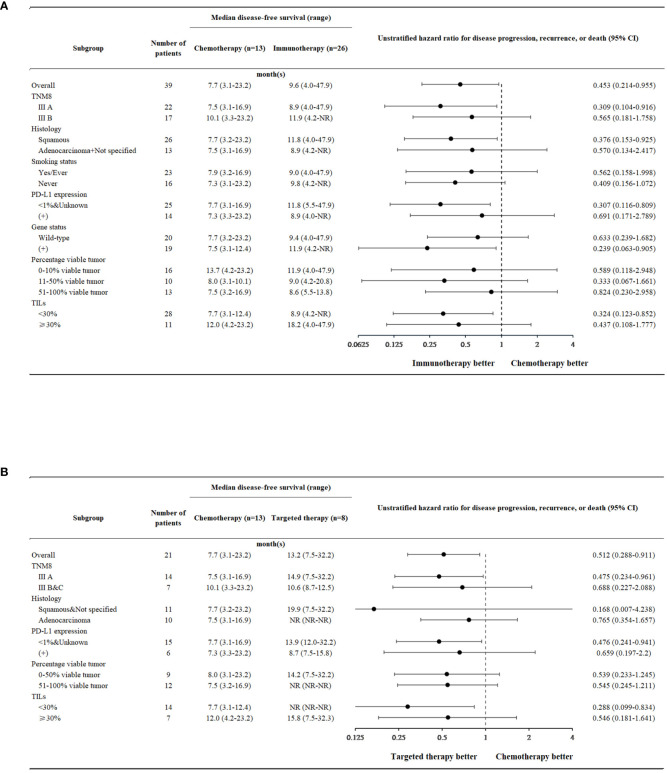
Forest plots. **(A)** Disease-free survival in neoadjuvant chemotherapy and neoadjuvant immunotherapy across different patient subgroups. **(B)** Disease-free survival in neoadjuvant chemotherapy and neoadjuvant targeted therapy across different patient subgroups.

In the majority of subgroup analyses, the immunotherapy arm or targeted therapy arm were found to exhibit superior disease-free survival compared with the chemotherapy arm (**Figure 4**).

Within the neoadjuvant immunotherapy arm, patients could be subdivided into the immunotherapy + chemotherapy group and the immunotherapy alone group (**Table 6**). Those who received immunotherapy + chemotherapy displayed a significantly better clinical response than those who received immunotherapy alone (P=0.031). Overall tumor regression rates (31.3 ± 26.2 vs. 6.0 ± 36.6) and pCR (21.7% vs. 0%) rates also favored the immunotherapy + chemotherapy group, though no significance was noted (P=0.098, 0.512). However, patients who were treated with immunotherapeutics alone seemed to have a longer disease-free survival [9.4 (4.0-NR) vs. 19.3 (8.6-47.9); P=0.185].

**Table 6:** Efficacy and survival in prescribed therapeutics subgroup from the neoadjuvant immunotherapy group and neoadjuvant targeted therapy arm.

	Immunotherapy + Chemotherapy n=26 (%)	Immunotherapy alone n=4 (%)	*P*-value
Clinical response	22 (84.6)	1 (25.0)	**0.031**
Clinical tumor regression from baseline (%),			
mean ± SD (range)	31.3 ± 26.2 (-14.7-100.0)	6.0 ± 36.6 (-45.7-38.3)	0.098
	n=23 (%)	n=3 (%)	
pCR	5 (21.7)	0 (0)	0.512
DFS, median (range)	9.4 (4.0-NR)	19.3 (8.6-47.9)	0.185^*^

^*^Mann-Whitney U test.

The bold values denote statistical significance at P<0.05 level.

The neoadjuvant immunotherapy group can also be further divided into the ‘PD-1 inhibitor’ group and the ‘PD-1 + CTLA-4 inhibitor’ group based on the immunotherapeutic agents administered to the patients. Patients who received PD-1 inhibitor along with CTLA-4 inhibitor seemed to have a longer DFS [28.3 (8.6-47.9) vs. 9.6 (4.0-NR)], but no better clinical response (50.0% vs. 78.6%) or pCR rate (0% vs. 20.8%). No significant differences were observed.

## 4 Discussion

Neoadjuvant therapy, particularly neoadjuvant immunotherapy, has exhibited phenomenal efficacy in phase II and III clinical trials, such as the LCMC3 trial, the NEOSTAR trial, the NADIM trial and the Checkmate 816 trial, and has been striving towards extended application in non-small cell lung cancer (9, 10, 13, 14). Currently, the primary challenge facing neoadjuvant therapy probably lies in identifying which therapy is both safe and effective. Prior study indicated that neoadjuvant immunotherapy tended to yield better pCR rates and curative effects than neoadjuvant chemotherapy and targeted therapy (15). In this interim analysis of study, we put emphasis on the safety and survival of different neoadjuvant treatments, exclusively in patients with resectable stage III (at baseline) NSCLC, and discovered a tendency towards better efficacy and longer survival without unmanageable toxicity or surgical difficulties in neoadjuvant immunotherapy and neoadjuvant targeted therapy compared with neoadjuvant chemotherapy.

The clinical response rates were highest in the targeted therapy arm and lowest in the chemotherapy arm. However, in the pathological setting, the immunotherapy arm ranked first, with a pCR rate of 19.2%. The slight incoherence between clinical and pathological response was probably due to the current RECIST 1.1 not being entirely suitable in the neoadjuvant setting, and new criteria might be necessary (14, 16). In this study, we chose to remain consistent with the initial criteria at baseline since there has been no consensus on this matter.

The term ‘surgical feasibility’ is one that holds objectivity for surgeons. Therefore, we subdivided surgical outcomes into minor categories like duration of surgery, amount of bleeding, number of drainage tubes, length of stay, etc., for the sake of quantification. Results seemed to favor targeted therapy, while it was similar between the immunotherapy and chemotherapy arms with regard to duration of stay, amount of bleeding, number of drainage tubes, lengths of stay, less invasive surgical approach (VATS) and so forth. These findings were consistent with those from the Checkmate 816 trial. Furthermore, clinical to pathological downstaging occurred mostly in patients receiving immunotherapy with(out) chemotherapy in our study. This was in line with the research conducted by Forde PM et al., which suggested that better response and a higher incidence of downstaging might, to some extent, related to the better surgical outcomes (10). However, this was not the case for neoadjuvant targeted therapy, as its rate of clinical to pathological downstaging was slightly lower. This could be attributed to the small sample size of our targeted therapy arm in comparison with the other two treatment arms.

As for adverse events, the situation was roughly the same amongst different treatment arms. Toxicity was overall manageable regardless of treatment or surgery related adverse events, and no new safety concerns were observed. These findings were consistent with previous studies that mainly focus on neoadjuvant immunotherapy, such as the NADIM trial, the Checkmate 816 trial and the NEOSTAR trial (8, 10, 13). The conclusions regarding neoadjuvant targeted therapy were quite similar according to Zhang Y et al. and Xiong L et al. The adverse events occurred in the erlotinib and GC chemotherapy arms of the EMERGING-CTONG 1103 trial were also among those most commonly seen with the treatment (7, 11, 17).

In the checkmate 816 trial involving patients with stage IB-IIIA resectable NSCLC, the median event-free survival was 31.6 months vs. 20.8 months with nivolumab plus chemotherapy and chemotherapy alone (P=0.005). The estimated one-year event-free survival rate was 76.1% vs. 63.4% (10). Zhang P et al. reported an 85.3% of disease-free survival in their study with sintilimab plus chemotherapy (18), while the 1-year EFS rate in Rothschild SI et al.’s research was 73% for durvalumab in addition to neoadjuvant chemotherapy (19). These studies all indicated that neoadjuvant immunotherapy led to a survival benefit. In our study with stage III NSCLC patients, the survival analysis also showed a significant difference between the chemotherapy arm and the immunotherapy arm (P=0.032).

The EMERGING-CTONG 1103 trial reported an improved progression-free survival (PFS) with erlotinib versus GC in IIIA N2 NSCLC patients (21.5 months vs. 11.4 months) (11), while Xiong L et al. found in a prospective, phase II single-arm study that IIIA N2 NSCLC patients treated with erlotinib had a 10.3-month median disease-free survival, and later suggested in another research that median PFS was 12.1 months vs. 11.0 months with erlotinib and cisplatin-based doublet chemotherapy (17, 20). Consistent with these findings, we discovered a significant improvement in DFS in the targeted therapy arm over the chemotherapy arm (P=0.015) as well.

These conclusions were also applicable to subgroup analysis, as the DFS was mostly in favor of immunotherapy and targeted therapy. However, no significance was noted between the immunotherapy and targeted therapy arms (P=0.500). There is little evidence suggesting differences between these two types of treatment in current studies, and further investigation is required in the future to determine the most effective therapy, or alternatively, what kind of patients are better suited for each treatment.

By the data cut-off date (March 4, 2023), 7 patients (14.9%) had died, so the overall survival of this cohort can only be analyzed in future studies. Given the single-center nature of this study with a limited sample size, potential bias and errors might have been present, which could only be avoided in future studies with larger cohorts. Moreover, the exclusion of patients who did not undergo surgery and lost to follow-up might generate potential bias. Lastly, we did not elaborate on the mechanisms behind each treatment, which should be a primary focus of future research. Despite all these limitations, our results demonstrated that in comparison with neoadjuvant chemotherapy, neoadjuvant immunotherapy or neoadjuvant targeted therapy could be a safe, effective and surgically feasible treatment option with tangible survival benefits for patients with resectable stage III NSCLC.

## 5 Conclusion

Generally speaking, neoadjuvant therapy appears to provide safe and effective results for stage III NSCLC patients, and does not typically pose any unmanageable perioperative issues. Compared with neoadjuvant chemotherapy, either neoadjuvant immunotherapy or neoadjuvant targeted therapy has a significantly longer disease-free survival and higher response rates (clinical and pathological). Surgical feasibility favored immunotherapy and targeted therapy over chemotherapy as well, and adverse events were similar across different treatment arms and overall manageable. Therefore, neoadjuvant immunotherapy and neoadjuvant targeted therapy could be promising for the treatment of patients with stage III NSCLC.

## Data availability statement

The original contributions presented in the study are included in the article/supplementary material. Further inquiries can be directed to the corresponding authors.

## Ethics statement

The studies involving human participants were reviewed and approved by the Institutional Review Board of Shanghai Chest Hospital. The patients/participants provided their written informed consent to participate in this study. Written informed consent was obtained from the individual(s) for the publication of any potentially identifiable images or data included in this article.

## Author contributions

Conceptualization: LG, SL and ZC; Methodology: LG, YY, SL and ZC; Data curation: YQ, LG and JS; Formal analysis: YQ; Writing - original draft preparation: YQ; Writing - review and editing: YQ and ZC; Investigation: YQ, JS and YZ; Project administration: SL and ZC; Funding acquisition: ZC; Resources: YQ and YZ; Software: YQ; Visualization: YQ; Supervision: YY, SL and ZC; Validation: YQ, LG, JS, YY, YZ, SL and ZC. All authors read and approved the final manuscript.

## References

[1] Siegel RL Miller KD Wagle NS Jemal A. Cancer statistics, 2023. CA Cancer J Clin (2023) 73(1):17–48. doi: 10.3322/caac.21763 36633525

[2] Thai AA Solomon BJ Sequist LV Gainor JF Heist RS. Lung cancer. Lancet (2021) 398(10299):535–54. doi: 10.1016/s0140-6736(21)00312-3 34273294

[3] Amin MB Edge SB Greene FL Byrd DR Brookland RK Washington MK. AJCC cancer staging manual Vol. XVII. Philadelphia: Springer Cham (2017). p. 1032.

[4] Daly ME Singh N Ismaila N Antonoff MB Arenberg DA Bradley J. Management of stage III non-small-cell lung cancer: ASCO guideline. J Clin Oncol (2022) 40(12):1356–84. doi: 10.1200/jco.21.02528 34936470

[5] Gentzler RD Riley DO and Martin LW. Striving toward improved outcomes for surgically resectable non-small cell lung cancer: the promise and challenges of neoadjuvant immunotherapy. Curr Oncol Rep (2020) 22(11):109. doi: 10.1007/s11912-020-00969-w 32803520

[6] Dudek AZ Lesniewski-Kmak K Larson T Dragnev K Isaksson R Gupta V. Phase II trial of neoadjuvant therapy with carboplatin, gemcitabine plus thalidomide for stages IIB and III non-small cell lung cancer. J Thorac Oncol (2009) 4(8):969–75. doi: 10.1097/JTO.0b013e3181add877 19633472

[7] Zhang Y Fu F Hu H Wang S Li Y Hu H. Gefitinib as neoadjuvant therapy for resectable stage II-IIIA non-small cell lung cancer: A phase II study. J Thorac Cardiovasc Surg (2021) 161(2):434–442.e2. doi: 10.1016/j.jtcvs.2020.02.131 32340810

[8] Provencio M Nadal E Insa A García-Campelo MR Casal-Rubio J Dómine M. Neoadjuvant chemotherapy and nivolumab in resectable non-small-cell lung cancer (NADIM): an open-label, multicentre, single-arm, phase 2 trial. Lancet Oncol (2020) 21(11):1413–22. doi: 10.1016/s1470-2045(20)30453-8 32979984

[9] Chaft JE Oezkan F Kris MG Bunn PA Wistuba II Kwiatkowski DJ. Neoadjuvant atezolizumab for resectable non-small cell lung cancer: an open-label, single-arm phase II trial. Nat Med (2022) 28(10):2155–61. doi: 10.1038/s41591-022-01962-5 PMC9556329 36097216

[10] Forde PM Spicer J Lu S Provencio M Mitsudomi T Awad MM. Neoadjuvant nivolumab plus chemotherapy in resectable lung cancer. N Engl J Med (2022) 386(21):1973–85. doi: 10.1056/NEJMoa2202170 PMC9844511 35403841

[11] Zhong WZ Chen KN Chen C Gu CD Wang J Yang XN. Erlotinib versus gemcitabine plus cisplatin as neoadjuvant treatment of stage IIIA-N2 EGFR-mutant non-small-cell lung cancer (EMERGING-CTONG 1103): A randomized phase II study. J Clin Oncol (2019) 37(25):2235–45. doi: 10.1200/jco.19.00075 31194613

[12] Hendry S Salgado R Gevaert T Russell PA John T Thapa B. Assessing tumor-infiltrating lymphocytes in solid tumors: A practical review for pathologists and proposal for a standardized method from the international immuno-oncology biomarkers working group: part 2: TILs in melanoma, gastrointestinal tract carcinomas, non-small cell lung carcinoma and mesothelioma, endometrial and ovarian carcinomas, squamous cell carcinoma of the head and neck, genitourinary carcinomas, and primary brain tumors. Adv Anat Pathol (2017) 24(6):311–35. doi: 10.1097/pap.0000000000000161 PMC5638696 28777143

[13] Cascone T William WN Jr. Weissferdt A Leung CH Lin HY Pataer A. Neoadjuvant nivolumab or nivolumab plus ipilimumab in operable non-small cell lung cancer: the phase 2 randomized NEOSTAR trial. Nat Med (2021) 27(3):504–14. doi: 10.1038/s41591-020-01224-2 PMC8818318 33603241

[14] Provencio M Serna-Blasco R Nadal E Insa A García-Campelo MR Casal Rubio J. Overall Survival and Biomarker Analysis of Neoadjuvant Nivolumab Plus Chemotherapy in Operable Stage IIIA Non-Small-Cell Lung Cancer (NADIM phase II trial). J Clin Oncol (2022) 40(25):2924–33. doi: 10.1200/jco.21.02660 PMC9426809 35576508

[15] Gu L Wang X Sun Y Xu Y Niu X Zhao R. An open, observational, three-arm clinical study of 2-3 cycles of treatment as neoadjuvant therapy in operable locally advanced non-small cell lung cancer: An interim analysis. Front Immunol (2022) 13:938269. doi: 10.3389/fimmu.2022.938269 36059450 PMC9437422

[16] Forde PM Chaft JE Smith KN Anagnostou V Cottrell TR Hellmann MD. Neoadjuvant PD-1 blockade in resectable lung cancer. N Engl J Med (2018) 378(21):1976–86. doi: 10.1056/NEJMoa1716078 PMC6223617 29658848

[17] Xiong L Li R Sun J Lou Y Zhang W Bai H. Erlotinib as neoadjuvant therapy in stage IIIA (N2) EGFR mutation-positive non-small cell lung cancer: A prospective, single-arm, phase II study. Oncologist (2019) 24(2):157–e64. doi: 10.1634/theoncologist.2018-0120 30158288 PMC6369937

[18] Zhang P Dai J Sun F Xia H He W Duan L. Neoadjuvant sintilimab and chemotherapy for resectable stage IIIA non-small cell lung cancer. Ann Thorac Surg (2022) 114(3):949–58. doi: 10.1016/j.athoracsur.2022.01.039 35176262

[19] Rothschild SI Zippelius A Eboulet EI Savic Prince S Betticher D Bettini A. SAKK 16/14: durvalumab in addition to neoadjuvant chemotherapy in patients with stage IIIA(N2) non-small-cell lung cancer-A multicenter single-arm phase II trial. J Clin Oncol (2021) 39(26):2872–80. doi: 10.1200/jco.21.00276 34251873

[20] Xiong L Lou Y Bai H Li R Xia J Fang W. Efficacy of erlotinib as neoadjuvant regimen in EGFR-mutant locally advanced non-small cell lung cancer patients. J Int Med Res (2020) 48(4):300060519887275. doi: 10.1177/0300060519887275 31885349 PMC7607055

